# Antimicrobial, Antioxidant, and Anticancer Activities of Some Novel Isoxazole Ring Containing Chalcone and Dihydropyrazole Derivatives

**DOI:** 10.3390/molecules25051047

**Published:** 2020-02-26

**Authors:** Afzal Shaik, Richie R. Bhandare, Kishor Palleapati, Srinath Nissankararao, Venkata Kancharlapalli, Shahanaaz Shaik

**Affiliations:** 1 Department of Pharmaceutical Chemistry Vignan Pharmacy College, Jawaharlal Nehru Technological University, Vadlamudi-522213, Andhra Pradesh, India; 2 Department of Pharmaceutical Sciences, College of Pharmacy & Health Sciences, Ajman University, Ajman PO Box 346, UAE; 3 Department of Pharmacognosy and Pharmaceutical Chemistry ASN Pharmacy College, Acharya Nagarjuna University, Tenali-522201, Andhra Pradesh, India; kishorpharmcog@gmail.com (K.P.); asnpctenali@gmail.com (V.K.); 4 Montvale, New Jersey, NJ 07645, USA; srinath.n2k@gmail.com; 5 Department of Pharmaceutical Chemistry Victoria College of Pharmacy, Acharya Nagarjuna University, Nallapadu-522001, Guntur District, Andhra Pradesh, India; shahanaazchemistry@gmail.com

**Keywords:** isoxazole, chalcones, dihydropyrazole, antibacterial activity, antifungal activity, anticancer activity

## Abstract

Our previous work identified isoxazole-based chalcones and their dihydropyrazole derivatives as two important five-membered heterocycles having antitubercular activity. Hence, in the present study, we biologically evaluated 30 compounds, including 15 isoxazole ring-containing chalcones (**17**–**31**) and 15 dihydropyrazoles (**32**–**46**) derived from these chalcones for their antimicrobial, antioxidant, and anticancer activities. Chalcones exhibited superior antibacterial and antioxidant activities compared to dihydropyrazoles. Among the chalcones, compound **28** showed potent antibacterial (MIC = 1 µg/mL) and antioxidant activities (IC_50_ = 5 ± 1 µg/mL). Dihydropyrazoles, on the contrary, demonstrated remarkable antifungal and anticancer activities. Compound **46** (IC_50_ = 2 ± 1 µg/mL) showed excellent antifungal activity whereas two other dihydropyrazoles **45** (IC_50_ = 2 ± 1 µg/mL) and **39** (IC_50_ = 4 ± 1 µg/mL) exhibited potential anticancer activity. The compounds were also tested for their toxicity on normal human cell lines (LO2) and were found to be nontoxic. The active compounds that have emerged out of this study are potential lead molecules for the development of novel drugs against infectious diseases, oxidative stress, and cancer.

## 1. Introduction

Heterocyclic compounds are a class of cyclic organic compounds containing heteroatoms, like nitrogen, sulfur, oxygen, etc., along with the carbon framework. The heterocyclic compounds possess diverse pharmacological activities and are employed in the treatment of a variety of diseases. Most of the therapeutic agents employed in the present-day therapy contain heterocyclic ring as the major structural component. Among these compounds, nitrogen-containing heterocyclic rings are distinctive not only because of their ease of synthesis but also due to their widespread distribution and biological profiles. Chalcones are open chain flavonoids containing the reactive propenone linker connected to two aryl rings. A literature survey revealed that chalcones and nitrogen-containing heterocycles, i.e., isoxazoles and dihydropyrazoles, possess a broad spectrum of biological activities, like antimicrobial, antioxidant, anticancer, antimalarial, antidepressant, antihistaminic, antitubercular, and anti-inflammatory [1,2,3,4,5,6,7,8,9,10,11,12,13,14,15,16,17,18,19,20,21,22,23,24,25,26,27,28,29,30,31,32,33,34,35,36,37,38,39,40,41,42,43,44,45,46,47,48].

**Figure 1:**
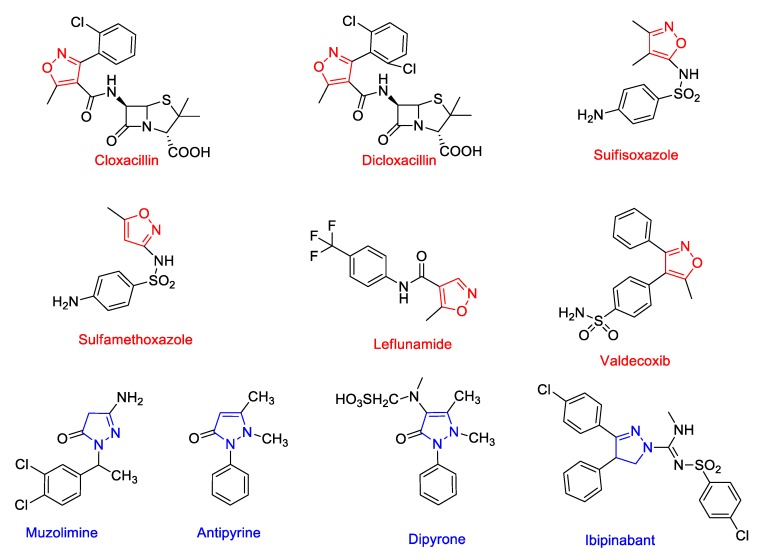
Structures of some clinically useful drugs containing isoxazole and dihydropyrazole rings.

Isoxazole, a five-membered heterocyclic ring, is present in therapeutic drugs, including the *β*-lactam antibiotics, cloxacillin and dicloxacillin, sulfisoxazole and sulfamethoxazole (antibacterials), valdecoxib (selective COX-II inhibitor), and leflunomide, an immunosuppressive disease-modifying antirheumatic drug [DMARD]. The completely reduced form of isoxazole, i.e., isoxazoline and isoxazolidine scaffolds, are seen in the antifungal agent drazoxolon and antitubercular antibiotic cycloserine, respectively. Dihydropyrazole is another interesting heterocyclic compound with two nitrogen atoms present in the adjacent positions of a five-membered ring. This ring can be conveniently synthesized from *α*, *β*-unsaturated carbonyl compounds by reacting with different kinds of hydrazines. The dihydropyrazole or its oxo derivative scaffold constitutes the part of drugs, including Muzolimine (Diuretic); Antipyrine, Aminopyrine, Ramifenazone, and Dipyrone (Analgesics); and Ibipinabant (anti-obesity cannabinoid receptor type 1 ((CB_1_) antagonist) (Figure 1).

**Figure 2:**
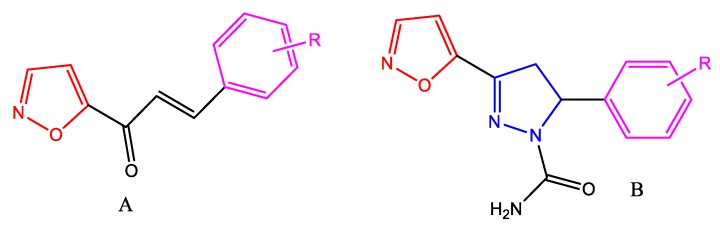
Previously designed isoxazole chalcone and dihydropyrazole derivatives.

Chalcones, a privileged structure in medicinal chemistry [6], are a type of open chain flavonoids bearing two aryl rings connected through a three-carbon spacer, the propenone linkage. The reactive *α*, *β*-unsaturated propenone fragment is not only responsible for the bioactivities but is also useful for the conversion of chalcones to different classes of heterocyclic compounds. There is a difference in the intensity of the bioactivities shown by chalcones due to variation in the nature and type of aryl rings. A literature survey on chalcones revealed that compounds containing heteroaromatic rings in their aryl portion showed excellent biological profiles [7,8]. We have previously reported heterocyclic ring-appended chalcones exhibiting antitubercular, antifungal, and cytotoxic activities [9,10,11,12,13]. Based on a literature survey [1,2,3,4,5,14,15,16,17,18,19,20,21,22,23,24,25,26,27,28,29,30,31,32,33,34,35,36,37,38,39,40,41,42,43,44,45,46,47,48] and our previously conducted studies [9,10,11,12,13], we herein report a continuation of that work, with a goal of further evaluating isoxazole-containing chalcones and its dihydropyrazole derivatives (privileged heterocyclic compounds) (Figure 2) for their antibacterial, antifungal, antioxidant, and anticancer activities. The results of these efforts and the structure–activity relationship for the series of isoxazole chalcones and dihydropyrazole derivatives are discussed here.

## 2. Results and Discussion

### 2.1. Chemistry

**Scheme 1:**
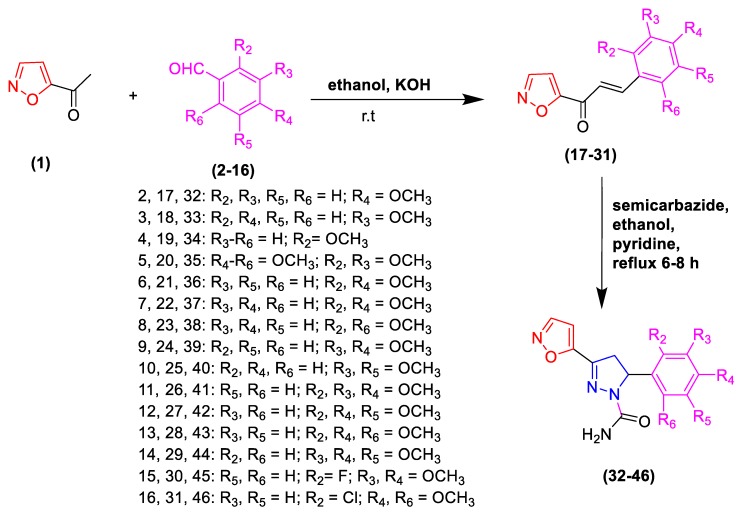
Synthesis of isoxazolylchalcones (**17**–**31**) and dihydropyrazoles (**32**–**46**).

The synthesis and characterization of compounds **17**–**31** and **32**–**46** has been published previously [9,11]. Chalcones (**17**–**31**) were prepared by Claisen–Schmidt condensation reaction (Scheme 1) in the presence of aqueous potassium hydroxide as a catalyst. The 1-(isoxazole-5-yl)ethanone (**1**) was condensed with substituted benzaldehyde (**2**–**16**) under basis conditions at room temperature for 24 h. The reaction was stopped by transferring the mixture on ice. The crude precipitate was recrystallized using chloroform to afford chalcones **17**–**31**. The target dihydropyrazoles (**32**–**46**) were obtained by treating the synthesized chalcones with semicarbazide in a catalytic amount of pyridine and purified using silica gel column chromatography.

### 2.2. Biological Studies

#### 2.2.1. Antibacterial and Antifungal Activities

Previously published studies suggested that chalcones/heteroarylchalcones and compounds having dihydropyrazoles exhibited antimicrobial, antioxidant, and cytotoxic activity [1,2,3,4,5,14,15,16,17,18,19,20,21,22,23,24,25,26,27,28,29,30,31,32,33,34,35,36,37,38,39,40,41,42,43,44,45,46,47]. Hence, we decided to screen our compounds for antibacterial, antifungal, antioxidant, and anticancer (prostrate cell line) activities. The target chalcones (**17**–**31**) and pyrazolines (**32**–**46**) were tested for their antimicrobial activities by serial tube dilution method against two types of bacterial and fungal strains (Table 1 and Table 2). The bacterial strains used for the testing, including Gram-positive *Staphylococcus aureus* and Gram-negative *Pseudomonas aeruginosa*, whereas the fungal strains employed were *Aspergillus niger* and *Candida tropicalis*.

Chalcones exhibited excellent antibacterial potency whereas the dihydropyrazoles showed tremendous antifungal activity compared to the standard drugs ciprofloxacin and fluconazole. In chalcones and dihydropyrazoles, the nature and position of the substituents on the phenyl ring played a crucial role for the antimicrobial activity. Compounds **17**–**29** had an electron-donating group (-OCH_3_) substituted at positions 2–6 on the aromatic ring and compounds **30** and **31** had electron-withdrawing groups (F, Cl) substituted at position 2 and electron-donating groups at position 3–6 on the aromatic ring. Compounds **17**–**19** represent monosubstituted chalcone derivative; **20**–**25** represent disubstituted chalcone derivative and compounds **26**–**31** represent trisubstituted chalcone derivative. In the monosubstituted chalcone series **17**–**19**, the methoxy group at position 2 and 4 showed better antibacterial and antifungal activity than at position 3 but lower than the standard drugs. Among the disubstituted chalcone series **20**–**25**, substitution at positions 2 and 6 showed better antibacterial activity than at positions 2,3 (**20**); 2,4 (**21**); 2,5 (**22**); 3,4 (**24**); and 3,5 (**25**). However, these compounds had lower activity than the standard drugs. In the trisubstituted series, **28** containing a 2,4,6-trimethoxyphenyl ring was the most potent (MIC 1 µg/mL) among the chalcone series and its antibacterial activity was found to be greater than ciprofloxacin. In contrast, the antifungal potency of **28** was found to be 2 µg/mL, which was less compared to fluconazole (1 µg/mL). On comparing compounds **17**, **19**, **23**, and **28**, it can be observed that the methoxy substituent is favorable to be substituted at positions 2 or 4 (**17** and **19**), 2 and 6 (**23**), or 2, 4 and 6 (**28**). Compound **31** containing 2-chloro-4,6-dimethoxyphenyl was next in potency to **28** with respect to antibacterial and antifungal activities. On comparing compounds **28** and **31**, it can be seen that substituting the electron-donating group instead of the electron-withdrawing group at position 2 was favorable for their antibacterial and antifungal activities. Most of the chalcones demonstrated moderate antibacterial and antifungal activity, with the minimum inhibitory concentration (MIC) ranging from 4–64 µg/mL. Some of the compounds viz., **18**, **25**, **26**, and **29** showed poor activity, with the MIC ranging between 64 and 256 µg/mL. It can be concluded from the structure–activity relationship studies that the presence of additional methoxy groups at the ortho and para positions (2, 4, and 6) was critical for the antimicrobial activity whereas substitution of methoxy groups in meta position (3) was deleterious for the antimicrobial and antifungal potency.

Dihydropyrazoles **32**–**46** exhibited better antifungal than antibacterial activity. As observed in the previous chalcone series, the nature and position of the substituents on the phenyl ring played a crucial role for the antifungal activity. Compounds **32**–**44** had an electron-donating group (-OCH_3_) substituted at positions 2-6 on the aromatic ring and compounds **45** and **46** had electron-withdrawing groups (F, Cl) substituted at positions 2 and electron-donating groups at positions 3-6 on the aromatic ring. Compounds **32**–**34**, **35**–**40**, and **41**–**46** represent monosubstituted, disubstituted, and trisubstituted dihydropyrazole derivatives. In the monosubstituted dihydropyrazole series, the substitution at position 4 (**32**) was found to be better than position 2 (**34**) for the antifungal activity (MIC 4 vs. 8 µg/mL). Methoxy substitution at position 3 (**33**) resulted in poor antibacterial and modest antifungal activity. Among the disubstituted dihydropyrazole series **35**–**40**, substitution at positions 2 and 6 (**38**) showed better antifungal activity (MIC 2 µg/mL) than at positions 2,3 (**35**); 2,4 (**36**); 2,5 (**37**); 3,4 (**39**); and 3,5 (**40**). However, compound **38** had lower antifungal activity than the standard drug fluconazole (MIC 1 µm/mL). In the trisubstituted series, compound **43** (2 µg/mL) did not show any marked increase in antifungal activity compared to compound **38** (2 µg/mL). Compound **43**, however, had an 8- and 4-fold improvement in the antibacterial activity compared to compound **38**, which indicated that the methoxy group at position 4 benefitted the antibacterial over antifungal activity. The trisubstituted series also had the presence of electron-donating and -withdrawing groups (**45** and **46)**. Structure activity relationship studies (SAR) studies suggested that halogen substituents on the phenyl ring (**45** and **46**) exhibited brilliant antifungal activity compared to fluconazole and improved their antibacterial activity by 8-fold for **45** (MIC 8µg/mL) vs. **41** (MIC 64 µg/mL) but was lowered for **46** (MIC 8 µg/mL) vs. **43** (MIC 4 µg/mL). The antifungal activity of compounds **45** and **46** was found to be the best in this series and better than the standard drug fluconazole. In general, the antibacterial potency of dihydropyrazoles was found to be less than that of chalcones (**43** MIC 4 µg/mL vs. **28** 1 µg/mL).

#### 2.2.2. Antioxidant Activity

The antioxidant activity of all 30 compounds was assessed using 1,1-diphenyl-2-picrylhydrazine (DPPH) free radical scavenging assay and the results are the average of three independent experiments (Table 3). Gallic acid was employed as a positive control. Both isoxazole-based chalcones and dihydropyrazole derivatives exhibited significant antioxidant activity. In the chalcone monosubstituted series (**17**, **18**, and **19**), substituting at positions 2 or 4 or 6 on the phenyl ring did not improve the antioxidant activity. In the disubstituted series (**20**–**25**), compound **25** with substitutions at position 3 and 5 on the phenyl ring gave the highest activity of 9 µg/mL. In the trisubstituted series (**26**–**31**), substitutions at positions 2, 4, and 6 resulted in the highest IC_50_ of 5 µg/mL among all the series. Replacement of the methoxy group (**26** and **28**) with either F (**30**) or Cl (**31**) resulted in a drop in the antioxidant activity, which suggested that electron-donating groups are favored at positions 2 on the phenyl ring. A similar phenomenon was observed in the dihydropyrazole series (**32**–**46**). Compound **43** was found to have an IC_50_ of 6 µg/mL and was the most potent in this series. However, its activity was found to be less than the standard gallic acid (IC_50_ = 5 µg/mL) It can be concluded from the chalcone and dihydropyrazole data that the antioxidant activity was found to be improved on the substitution of the electron-donating group (-OCH_3_) on the phenyl ring at positions 2, 4, and 6. In the chalcone series, compound **28** containing three methoxy groups at positions 2, 4, and 6 was the most potent of the 30 compounds and its activity was equal to that of the standard, having an IC_50_ 5 µg/mL.

#### 2.2.3. Anticancer Activity

All the 30 compounds were evaluated for their anticancer potency against the prostate cancer cell line, **DU-145**, employing colorimetric tetrazolium dye (MTT) assay. In the chalcone monosubstituted series **17**–**19**, substituting at positions 2, 4, and 6 did not improve the anticancer activity. Among the disubstituted series **20**–**25**, substitution at position 3 and 4 (compound **24**) produced the best activity but was lower than the standard. Among the trisubstituted series **26**–**31**, having an electron-withdrawing (F) and -donating groups (-OCH_3_) at position 2, 3, and 4 (compound **30**) resulted in the highest potency among the chalcone series. This suggests that electron-withdrawing and -donating groups on the aromatic ring were required for improving the potency among the chalcone series. Compound **30**, the most potent chalcone, was equipotent to the standard Docetaxel at IC_50_ 5 ± 1 µg/mL. Similar phenomena can be seen among the dihydropyrazole series. In the monosubstituted series **32**–**34**, substituting position 2, 4, or 6 did not yield any improvement in the activity. In the disubstituted series **35**–**40**, compound **39** having electron-donating substitution at 3 and 4 was found to be the most potent, with IC_50_ 4 ± 2 µg/mL. In the trisubstituted series **41**–**46**, compound **45** was found to be the most potent having fluoro at position 2 and –OCH_3_ at positions 3 and 4, having an IC_50_ of 2 ± 1 µg/mL. It can be seen that the dihydropyrazole derivatives exhibited superior activity than the chalcones **39** vs. **24** and **45** vs. **30** (Table 4). The SAR features indicated that heterocyclic pyrazoline ring was more essential than the propenone motif found in chalcones. The activity of compounds **39** and **45** was greater than the standard, Docetaxel (MIC = 5 µg/mL). Chalcone **30** and dihydropyrazole **44** exhibited equipotent activity as that of the standard. The compounds **24**, **29**, **41**, and **26** were next in activity, with IC_50_ values of 6, 8, 9, and 10 µg/mL, respectively. All the other compounds exhibited modest anticancer activity, with IC_50_ values ranging from 11–32 µg/mL. The compounds were also tested against the human normal cells (**L02**) and were found to be nontoxic to these cells.

**Figure 3:**
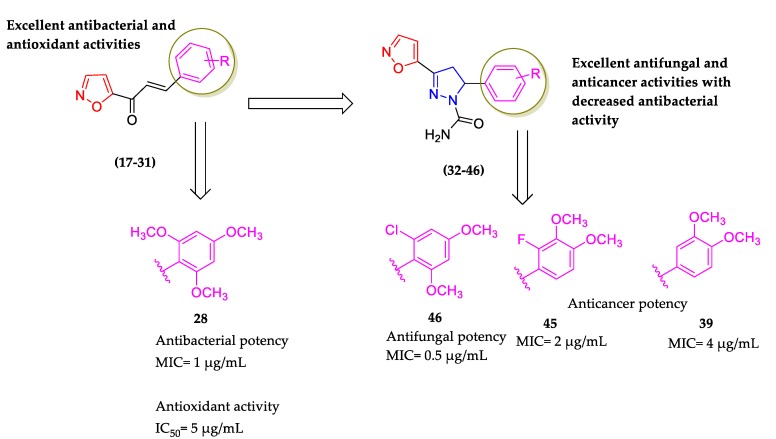
Summarization of the antibacterial, antifungal, antioxidant, and cytotoxic activities of chalcones (**17**–**31**) and dihydropyrazoles (**32**–**46**).

A summary of the antibacterial, antifungal, antioxidant, and cytotoxic activity of chalcones (**17**–**31**) and dihydropyrazoles (**32**–**46**) is depicted in Figure 3.

## 3. Materials and Methods

### 3.1. Biological Activity Studies

#### 3.1.1. Antibacterial and Antifungal Activities

The antibacterial and antifungal activity of the novel chalcones (**17** to **31**) and dihydropyrazoles (**32** to **46)** against the selected bacterial and fungal strains was assessed by following the procedure described in our previous paper [10].

#### 3.1.2. Antioxidant Activity

The use of the DPPH assay provides an easy and rapid way to evaluate antioxidants by spectrophotometry, hence it can be useful to assess various products at a time. The purpose of this study was to evaluate the antioxidant activity of chalcones and pyrazolines using the DPPH free radical assay. The percentage of antioxidant activity (AA%) of all the compounds was assessed by the DPPH free radical assay. The measurement of the DPPH radical scavenging activity was performed according to methodology described by Brand-Williams et al. The samples were reacted with the stable DPPH radical in an ethanol solution. A 0.1 mM solution of DPPH was prepared by dissolving DPPH in methanol. Gallic acid was taken as a reference standard and different concentrations of test samples (5–100 µg/mL) and standard (1.0, 2.5, and 5.0 µg/mL) were prepared using methanol. One milliliter of 0.1 mM DPPH solution was added to 3 mL of all concentrations of test samples and standard separately. These mixtures were kept in dark for about 30 min and the absorbance was measured at 517 nm [49]. The capability to scavenge the DPPH radical was calculated using the formula:

(1) $$\mathrm{DPPH}\;\mathrm{scavanged}\;\left(\%\right)=\;\frac{\mathrm{Absorbance}\;\mathrm{of}\;\mathrm{control}-\mathrm{Absorbance}\;\mathrm{of}\;\mathrm{sample}}{\mathrm{Absorbance}\;\mathrm{of}\;\mathrm{control}}*100$$

when DPPH reacted with an antioxidant compound and was reduced, the change in color (from deep violet to light yellow) was read [Absorbance (Abs)] at 517 nm after 100 min of reaction using a UV-VIS spectrophotometer.

#### 3.1.3. Anticancer Activity

The in vitro anticancer activity of chalcones (**17** to **31**) and dihydropyrazoles (**32** to **46**) was evaluated by the Mosmann’s MTT assay as described previously [34].

## 4. Conclusions

In a nutshell, we reported the antibacterial, antifungal, antioxidant, and anti-prostate cancer and structure–activity relationship studies of 30 isoxazole and substituted phenyl ring-containing compounds, including 15 chalcones and dihydropyrazoles. All the compounds were found to be nontoxic against the human normal cell line LO2. Biological screening data indicated that chalcones exhibited excellent antibacterial and antioxidant activities whereas the dihydropyrazole derivatives showed superior antifungal and anticancer activities. It was observed that the electronic property (electron withdrawing and electron releasing) of the substituents on the phenyl ring was instrumental for the difference in the potency of the compounds. For instance, chalcone **28** containing the 2,4,6-trimethoxy phenyl ring showed the potent antibacterial activity as well as antioxidant activity whereas dihydropyrazole **46** bearing the 2-chloro-4,6-dimethoxyphenyl was the potent antifungal compound and **45** and **39** having 2-fluoro-3,4-dimethoxyphenyl and 3,4-dimethoxyphenyl substituents showed excellent anticancer activity against the prostate cancer (DU-145) cell line. These findings have identified potential lead molecules against the proposed activities. Further studies are under progress to assess the influence of the bioisosteric replacement of the isoxazole scaffold with other privileged five-membered heterocyclic rings.

## Figures and Tables

**Table 1:** Results of the antibacterial and antifungal activities of chalcones (MIC µg/mL; **17**–**31**).

Compound	*S aureus*	*P aeruginosa*	*A niger*	*C tropicalis*
**17**	4	8	8	8
**18**	128	128	256	256
**19**	4	4	8	16
**20**	32	64	64	32
**21**	2	4	8	16
**22**	32	32	64	64
**23**	4	4	4	4
**24**	64	32	128	64
**25**	256	256	256	512
**26**	64	64	128	128
**27**	16	16	32	32
**28**	1	1	2	2
**29**	64	128	128	256
**30**	4	4	16	16
**31**	2	2	4	4
Ciprofloxacin	2	2	---	---
Fluconazole	---	---	1	1

**Table 2:** Results of the antibacterial and antifungal activity of dihydropyrazoles (MIC µg/mL; **32**–**46**).

Compound	*S aureus*	*P aeruginosa*	*A niger*	*C tropicalis*
**32**	16	16	4	4
**33**	128	128	64	64
**34**	16	16	8	8
**35**	64	64	16	16
**36**	8	8	4	8
**37**	64	32	32	32
**38**	32	16	2	2
**39**	128	64	32	32
**40**	256	256	64	64
**41**	64	128	64	64
**42**	32	16	16	16
**43**	4	4	2	2
**44**	64	256	16	16
**45**	8	8	0.5	1
**46**	8	8	0.5	0.5
Ciprofloxacin	2	2	---	---
Fluconazole	---	---	1	1

**Table 3:** Comparison of the DPPH assay results of chalcones (**17**–**31**) and dihydropyrazoles (**32**–**46**) (IC_50_ values in µg/mL).

Compound	IC_50_	Compound	IC_50_
**17**	33 ± 1	**32**	44 ± 1
**18**	38 ± 2	**33**	51 ± 2
**19**	32 ± 2	**34**	38 ± 2
**20**	24 ± 1	**35**	32 ± 1
**21**	18 ±2	**36**	25 ± 2
**22**	26 ±2	**37**	39 ± 2
**23**	16 ±1	**38**	28 ± 1
**24**	29 ±1	**39**	49 ± 1
**25**	9 ± 1	**40**	12 ± 1
**26**	6 ± 1	**41**	8 ± 1
**27**	7 ± 2	**42**	10 ± 2
**28**	5 ± 1	**43**	6 ± 1
**29**	8 ± 2	**44**	12 ± 2
**30**	45 ± 2	**45**	55 ± 2
**31**	48 ± 1	**46**	62 ± 1
Gallic acid 5 ± 1			

**Table 4:** Comparison of the MTT assay anticancer results of chalcones (**17**–**31**) and dihydropyrazoles (**32**–**46**) (IC_50_ values in µg/mL).

Compound	DU-145	Human Normal Cells (L02)	Compound	DU-145	Human Normal Cells (L02)
**17**	32 ± 2	>40	**32**	28 ± 1	>40
**18**	20 ± 1	>40	**33**	18 ± 2	>40
**19**	36 ± 1	>40	**34**	32 ± 2	>40
**20**	22 ± 1	>40	**35**	16 ± 2	>40
**21**	29 ± 2	>40	**36**	26 ± 2	>40
**22**	38 ± 1	>40	**37**	31 ± 2	>40
**23**	33 ± 2	>40	**38**	20 ± 2	>40
**24**	6 ± 2	>40	**39**	4 ± 2	>40
**25**	12 ± 1	>40	**40**	8 ± 2	>40
**26**	10 ± 2	>40	**41**	9 ± 1	>40
**27**	26 ± 3	>40	**42**	18 ±2	>40
**28**	30 ± 2	>40	**43**	21 ± 2	>40
**29**	8 ± 2	>40	**44**	5 ± 2	>40
**30**	5 ± 2	>40	**45**	2 ± 1	>40
**31**	16 ±2	>40	**46**	12 ± 2	>40
Docetaxel 5 ± 1					

Data presented as mean ± SD (*n* = 3). All the compounds and the standard dissolved in DMSO, diluted with culture medium containing 0.1% DMSO. The control cells were treated with culture medium containing 0.1% DMSO.
